# ‘Grinning and bearing it’—A survey study exploring animal‐related injuries in UK and Irish veterinary students

**DOI:** 10.1002/vro2.70041

**Published:** 2026-07-10

**Authors:** Tamzin Furtado, Lois Kennedy, Gina Pinchbeck, John S. P. Tulloch

**Affiliations:** ^1^ Department of Livestock and One Health, Institute of Infection, Veterinary, and Ecological Sciences University of Liverpool Liverpool UK; ^2^ School of Veterinary Science, Institute of Infection, Veterinary, and Ecological Sciences University of Liverpool Liverpool UK

## Abstract

**Background:**

While veterinary surgeons are known to have particularly high rates of injury compared to other sectors, little is known about the rates of injury among veterinary students. This study aims to understand animal‐related injury rates, injury context and mechanisms, attitudes to reporting injuries, and resultant behaviour among UK and Irish veterinary students.

**Methods:**

A survey was distributed to students across all veterinary schools operating in the UK and Ireland in 2021. Questions explored participants’ experience of injury through asking about their most recent and most severe injuries via quantitative and free‐text questions. Data were analysed using descriptive statistics, logistic regression and qualitative content analysis.

**Results:**

Five hundred thirty‐three responses were included in the analyses. Overall, 47.5% of the students reported having been injured by an animal during the veterinary degree, and 35.5% of the students reported being injured within the last 12 months. Most recent injuries were caused by companion animals (38.0%), livestock (37.6%) and equids (23.5%). For their most severe injuries, 48.7% involved livestock, 28.7% companion animals and 22.1% equids. The content analysis highlighted that students normalised injuries and infrequently reported injuries to the university. It was very rare for students to take time off from their studies or placements due to course pressures.

**Conclusions:**

These findings reflect concerningly high levels of injury, which are being under‐reported and reflect a culture of injury acceptance and expectation among students. Veterinary schools should consider lessons learned in other work environments that have been successful in changing safety culture.

## INTRODUCTION

The veterinary profession has some of the highest levels of non‐fatal injuries: in 2023, the US Bureau of Labor Statistics recorded that ‘Veterinary services’ had the second highest non‐fatal injury incidence rate out of any industry, 10.6 injuries per 100 full‐time equivalents, around 38,000 cases annually,^1^ which is more than four times the national incidence rate of 2.5.

Collecting data regarding the incidence of non‐fatal injuries within the UK and Irish veterinary workforces is challenging, as the industry is so small that data are often not presented. Survey data have suggested that being an equine veterinarian was one of the most hazardous civilian professions due to severity and frequency of injuries,^2^ and that 58.3% of companion animal veterinarians,^3^ 48.6% of equine veterinarians and 62.5% of large animal veterinarians are injured at work annually.^4^ These injuries are predominately animal related.^3^, ^4^, ^5^, ^6^, ^7^, ^8^, ^9^, ^10^ US data suggest that animal‐related injuries make up 49.3% of all injuries in veterinarians that result in days away from work, a further 23.2% were due to falls, slips or trips.^1^

Research has indicated that 25%‒33% of injured UK equine veterinarians require hospital treatment,^2^, ^4^ while 16% of horse‐related injuries among UK veterinary school staff required hospital attendance.^5^ These injuries were predominately caused by kicks while performing a clinical examination, in particular distal limb examinations.^4^ In contrast, cattle‐related injuries, mainly kicks and crushes during a clinical examination, resulted in higher attendance at hospitals for medical treatment (21%‒40%).^4^, ^5^

While equine and bovine incidents are often more severe, dogs and cats account for a higher frequency of injuries, typically through bites and scratches.^3^, ^5^, ^6^ Beyond animal‐related injuries, the sector remains at risk from needlestick and sharp injuries, traffic accidents and ergonomic injuries.^3^, ^6^, ^11^, ^12^ The three most prevalent injuries resulting in days away from work or restricted activity are puncture wounds (48.6%), fractures (12.7%) and sprains (9.2%).^1^

There is emerging evidence that the profession has a complex relationship with work‐related injuries, with a culture that minimises the risk and consequences of injury and thus promotes injury acceptance as ‘just part of the job’,^3^, ^13^, ^14^ in particular the risk posed by animals.^15^ Many within the profession only identify something as a work‐related injury if it limits their ability to perform their job or that they have to attend a hospital.^14^ Given this context, it is important to understand whether these same injuries are prevalent for veterinary students, and whether the context and culture around them are similar. If they are similar, then veterinary schools provide the perfect place to challenge the cultural norms and to develop intervention and education programmes to minimise injury risk.

Several studies have provided insight into veterinary student physical injury. A 10‐year review of UK veterinary school injury reports found that students had lower levels of injuries than staff, and that injuries were predominantly associated with cats and dogs.^5^ In Australia, the most prevalent causes of injury to veterinary students were sharps injuries (40.0%), animal bites (39.6%) and being rammed/pushed over by a large animal.^6^ However, student data are likely skewed by significant under‐reporting; evidence suggests that students frequently under‐report zoonotic infections, meaning official figures may not be representative of true risk levels.^16^

In the UK and Republic of Ireland, the potential for injury is compounded by the diverse environments encountered during the 5‐year undergraduate curriculum. These can be within university premises as well as on external placements. These are called extra‐mural studies (EMS), and can be pre‐clinical in nature (i.e., on farm, riding schools and kennels) or clinical (i.e., veterinary practices).^17^, ^18^ It is unclear how these diverse environments might impact the risk of injury to veterinary students or accident reporting.

Given these concerning data and the paucity of contextual and behavioural analysis, further study is warranted. Considering that the main occupational hazard in the veterinary industry is animals, we decided to focus solely on animal‐related injuries. Thus, the aim of this study was to establish the prevalence of animal‐related injuries in UK and Irish veterinary students, to describe the context around these injuries, and to describe their consequences, both physically and behaviourally.

## MATERIALS AND METHODS

An online cross‐sectional survey was designed, including closed and open‐ended questions. Respondents were asked about personal demographics (i.e., sex and age) and details of their university, year of study and stage of course (i.e., pre‐clinical or clinical). They were subsequently asked about animal‐related injuries that they had acquired during their studies. This was followed by a series of more detailed questions about their most recent and their most severe injury. These items were chosen for clear reporting, in order to obtain information on injuries at a range of severities. Both sets of questions included items about where and how the injury was acquired, the species involved, medical consequences, student attitude to the injury, any resultant behaviour change and the reporting process.

The survey was piloted with 30 veterinary students and amended based on their feedback (these data were not included in analysis). It was purposively distributed through social media via accounts with large veterinary student followings, such as university veterinary societies. It was additionally distributed through email via official university webmail (Dublin, Edinburgh, Glasgow, Liverpool and Nottingham). Any current veterinary student in the UK and Ireland was eligible to participate. The survey was open from 8 July 2021 to 31 August 2021. To ensure statistical representativeness, a sample of size 365 was calculated using the finite population correction based on the estimated UK and Republic of Ireland veterinary student population (*e* = 0.05, *Z* = 1.96, *p* = 0.5).^19^

Demographic characteristics of respondents were described. Overall injury prevalence was calculated and stratified by demographic data. Logistic regression was performed to identify any association between demographic variables and reporting having acquired an injury during their time at veterinary school. Variables taken forward for multivariable analysis were selected through substantive knowledge and statistical significance, with multicollinearity checked during this process. The analysis of close‐ended questions related to the context of injury, attitude of injured person and reporting culture were carried out descriptively and stratified by the most prevalent species involved.

In the Republic of Ireland, under The Safety, Health and Welfare at Work (Reporting of Accidents and Dangerous Occurrences) Regulations 2016 (S.I. no. 370 of 2016), employers legally must report to the Health and Safety Authority (HSA) any work‐related injuries that result in an employee being unable to carry out normal work for more than three consecutive days.^20^ In the UK, under the Reporting of Injuries, Diseases and Dangerous Occurrences Regulations (RIDDOR), employees must report any ‘specified injury’ (which includes fractures, amputations, loss of consciousness, among others), or if they are unable to work for more than 7 days to the Health and Safety Executive (HSE).^21^ However, as veterinary students are not employees, there is neither legal requirement to report injuries to the HSE or HSA, nor to record time off studies. Thus, we have classified respondent‐reported injuries as RIDDOR reportable based solely on the list of specified injuries. The overall prevalence of these types of injuries was calculated and stratified by species.

Open‐ended questions with free‐text responses were analysed using an inductive content analysis.^22^, ^23^ Each response was initially read through, while notes around initial impression of content were created. Then, responses were considered in isolation and labelled with a code that reflected these categories. Codes were created in an iterative fashion and so were combined, revised and deleted as more responses were read. The final set of codes was generated by repetition of this process to ensure internal validity of content codes. These were then counted to facilitate a quantitative comparison of content. Due to the nature of this intended analysis, responses to each question were coded separately. However, overall themes were then compared across different questions and across species.

Data masking was used to protect personally identifiable information of students; this was performed through aggregation of data or redacting of quotes. This study received ethical approval from the University of Liverpool Veterinary Research Ethics Committee (VREC1103).

## RESULTS

There were 578 respondents to the survey; however, 7.8% (*n* = 45) responses were excluded from analysis as 29 individuals had incomplete demographic information, and 16 individuals answered no questions past the demographics section. This resulted in 533 survey responses available for analysis. The estimated veterinary student population in 2021 in the UK and Republic of Ireland was 7241.^19^ If all students were exposed to the survey, then the crude response rate was 7.4%. Respondents were predominately female, 18‒24 years old, of white ethnicity, British and with no self‐identified disability (Table 1). These same demographics were reflected in those that had been injured. Women were slightly over‐represented, as in 2021, 80% of UK veterinary graduates were female.^19^ Almost 80% of respondents were from five of the 10 universities where veterinary degrees could be obtained at the time of study. First‐year students were under‐represented.

**TABLE 1:** Demographics of veterinary students receiving animal‐related injuries, and univariable analysis exploring demographic factors associated with acquiring such an injury.

Demographic variables	Respondent characteristics (percent, 95% CI)	Percentage injured (percent, 95% CI)	Univariable analysis	
			Odds ratio of injury (95% CI)	*p*‐Value
Sex (*n* = 527)				
Female	89.8% (86.8‒92.2)	48.2% (43.6‒52.8)	Ref	
Male	10.2% (7.8‒13.2)	40.7% (27.6‒55.0)	0.74 (0.41‒1.30)	0.30
Age (*n* = 533)				
18‒24	81.8% (78.3‒85.0)	47.2% (42.5‒52.1)	Ref	
25‒29	15.4% (12.4‒18.7)	50.0% (38.8‒61.3)	0.74 (0.25‒2.10)	0.58
30+	2.8% (1.6‒4.6)	37.5% (15.2‒64.6)	1.12 (0.70‒1.79)	0.65
Ethnicity (*n* = 528)				
White	93.8% (91.3‒95.7)	46.7% (42.2‒51.2)	0.73 (0.35‒1.48)	0.38
All other ethnic groups combined	6.3% (4.3‒8.7)	54.5% (36.4‒71.9)	Ref	
Self‐identified disability (*n* = 525)				
No	90.7% (87.9‒93.0)	46.8% (42.3‒51.4)	Ref	
Yes	9.3% (7.0‒12.2)	53.1% (38.3‒67.5)	1.28 (0.71‒2.33)	0.41
Nationality (*n* = 533)				
British	77.5% (73.7‒81.0)	48.9% (44.0‒53.9)	Ref	
Irish	9.2% (6.9‒12.0)	34.7% (21.7‒49.6)	0.55 (0.29‒1.02)	0.06
American	4.5% (2.9‒6.6)	33.3% (15.6‒55.3)	0.52 (0.21‒1.21)	0.14
Canadian	2.4% (1.3‒4.1)	69.2% (38.6‒90.9)	2.35 (0.75‒8.78)	0.16
Other nationalities combined	6.4% (4.5‒8.8)	50.0% (32.4‒67.6)	1.04 (0.52‒2.11)	0.90
Degree characteristics				
University (*n* = 533)				
University of Liverpool	21.6% (18.2‒25.3)	49.6% (40.1‒59.0)	Ref	
University of Nottingham	17.4% (14.3‒20.9)	47.3% (36.9‒57.9)	0.91 (0.53‒1.58)	0.75
University of Bristol	15.6% (12.6‒18.9)	41.0% (30.3‒52.3)	0.71 (0.40‒1.25)	0.23
University College Dublin	13.1% (10.4‒16.3)	38.6% (27.3‒51.0)	0.64 (0.34‒1.16)	0.15
Royal Veterinary College	11.3% (8.7‒14.3)	56.7% (43.2‒69.4)	1.33 (0.71‒2.51)	0.37
University of Cambridge	5.8% (4.0‒8.2)	61.3% (42.2‒78.2)	1.61 (0.72‒3.70)	0.25
University of Surrey	5.4% (3.7‒7.7)	58.6% (38.9‒76.5)	1.44 (0.64‒3.35)	0.39
University of Edinburgh	4.7% (3.1‒6.8)	52.0% (31.3‒72.2)	1.10 (0.46‒2.65)	0.83
University of Glasgow	3.9% (2.5‒6.0)	38.1% (18.1‒61.6)	0.63 (0.23‒1.60)	0.34
Harper and Keele Veterinary School	1.1% (0.4‒2.4)	0%	Not calculable	
Year of study				
1	11.4% (8.9‒14.5)	18.0% (9.4‒30.0)	Ref	
2	20.1% (16.8‒23.7)	35.5% (26.5‒45.4)	2.50 (1.20‒5.58)	0.02
3	14.4% (11.6‒17.7)	50.6% (39.0‒62.2)	4.67 (2.17‒10.67)	<0.001
4	16.5% (13.5‒19.9)	54.5% (43.6‒65.2)	5.45 (2.58‒11.97)	<0.001
5	31.3% (27.4‒35.5)	55.1% (47.2‒62.8)	5.58 (2.80‒11.97)	<0.001
6	4.3% (2.8‒6.4)	78.3% (56.3‒92.5)	16.36 (5.33‒58.97)	<0.001
Newly graduated	1.9% (0.9‒3.4)	70.0% (34.8‒93.3)	N/A	
Stage of degree				
Pre‐clinical	35.8% (31.8‒40.1)	31.9% (25.4‒39.1)	Ref	
Clinical	64.2% (59.9‒68.2)	56.1% (50.7‒61.5)	2.73 (1.89‒3.97)	<0.001
Know the injury reporting protocol at their university (*n* = 533)				
Yes	42.6% (38.4‒46.9)	44.1% (37.5‒50.8)	Ref	
No	57.4% (53.1‒61.7)	49.3% (43.6‒55.1)	1.27 (0.90‒1.79)	0.17

Abbreviation: CI, confidence interval.

Gender was excluded from analysis as there were less than 10 individuals that identified as being non‐cis‐gendered and we did not want these individuals to be personally identifiable. All ethnic groups, except the white British group, were aggregated into the group ‘all other ethnic groups combined’ as there were less than 10 individuals in these groups. Twenty‐nine nationalities were represented, but 25 of these had less than 10 individuals and so these were aggregated into the group ‘other nationalities combined’. When asked about their academic year, 10 individuals wrote in the free text ‘New Graduate’, as the survey was distributed just after many students’ graduations, we included them for descriptive analysis as their experiences would relate to their time in veterinary education. However, they were excluded from the logistic regression.

Univariable analysis revealed that there was no association between any demographic variable or attendance of any one university and the odds of acquiring an injury (Table 1). The only significant associations were between year and ‘stage of the degree’ and injury acquisition. The longer a student had been enrolled in the course, the greater the odds of being injured, and students in their clinical years were more likely to have been injured than if they were in the pre‐clinical part of the course. Due to strong collinearity (*r* = 0.85) between the two univariable variables of interest (‘Year of Degree’ and ‘Stage of Degree’), a multivariable model was not created.

Overall, 47.5% (95% confidence interval [CI] 43.2‒51.8, *n* = 253) of students reported having been injured by an animal during the veterinary degree, 35.5% (95% CI 31.4‒39.7) of students reported being injured within the last 12 months. Of those injured, 71.1% (95% CI 65.1‒76.7) reported having 1‒3 injuries, 18.2% (95% CI 13.6‒23.5) 4‒5 injuries, 5.9% (95% CI 3.4‒9.6) 6‒9 injuries, and 3.6% (95% CI 1.6‒6.6) having 10 or more injuries.

Ninety‐five percent of responses (*n* = 233/245) had enough contextual information to conclude whether their most recent injury was of a level that would be reportable to HSE via RIDDOR if the student had been an employee. Five percent of recent injuries (5.2%, 95% CI 2.7‒8.8) were RIDDOR reportable. All these injuries were fractures, except one concussion. Ninety‐nine percent of responses (*n* = 195/196) had enough contextual information to conclude whether their most severe injury was RIDDOR reportable. Nine percent of most severe injuries (9.2%, 95% CI 5.6‒14.2) were RIDDOR reportable. All these injuries were fractures or concussions.

Seventeen different species were involved in the students’ most recent injuries, and 16 in their most severe injuries. Most recent injuries were caused by: companion animals (38.0%, 95% CI 31.8‒44.6), livestock (37.6%, 95% CI 31.4‒44.2), equids (23.5%, 95% CI 18.2‒29.5) and wild animals (0.9%, 95% CI 0.1‒3.1), with 87.8% linked to five species: horses, cattle, cats, dogs and sheep. For the most severe injuries, 48.7% (95% CI 41.5‒56.0) involved livestock, 28.7% (95% CI 22.5‒35.6) were companion animals, 22.1% (95% CI 16.4‒28.5) were equids and 0.5% (95% CI 0.0‒2.8) were wild animals, with 90.3% associated with the same five species.

Fifty percent (50.2%) of the most recent injuries occurred during the clinical part of the veterinary degree, 82.0% of injuries occurred on EMS placements, 15% of injured students sought medical treatment beyond first aid, and 18.8% took more than 2 weeks to physically recover, but the majority (99.0%) did not take any time off their studies to recover. Only 16.8% of students informed their veterinary school about the injury. For the most severe injuries, 38.7% occurred during the clinical part of the veterinary degree, 87.8% of injuries occurred on EMS placements, 18.1% of injured students sought medical treatment beyond first aid, 26.9% took more than 2 weeks to physically recover, and 98.4% took no time off their studies to recover. Only 12.8% of students informed their veterinary school about the injury.

### Livestock‐related injuries

Over one‐third of the most recent injuries and over 40% of the most severe injuries were associated with cattle and sheep. These overwhelmingly occurred in pre‐clinical years and on‐farm EMS placements (Table 2). Cattle‐related injuries predominately occurred while the students were involved in three key activities: drug administration (23.3% of recent cattle injuries and 24.2% of most severe), herding (21.4% and 15.2%) and milking (21.4% and 21.2%). More than one in five of these injuries occurred when the student was by themselves. Cattle‐related injuries were mainly the result of kicks (48.9% of recent and 47.1% of severe) and crushes (36.2% and 43.1%). The distribution of body parts injured was dispersed: hand (23.9% and 25.5%), leg (26.1% and 27.4%), arm (17.4% and 15.7%), foot (14.6% and 15.7%) and head (4.3% and 5.9%). The injuries were overwhelmingly bruises (76.6% and 68.6%), but fractures (8.5% and 11.8%) and concussion (2.0% of severe injuries) occurred. Six percent (6.4%) of recent cattle‐related injuries and 9.8% of severe injuries were RIDDOR reportable injuries.

**TABLE 2:** Context of injuries to veterinary students caused by livestock and horses.

	Recent cattle injuries (*n* = 48), 20.4% of the most recent injuries (percent, 95% CI)	Severe cattle injuries (*n* = 51), 26.2% of the most severe injuries (percent, 95% CI)	Recent sheep injuries (*n* = 32), 13.6% of the most recent injuries percent, 95% CI)	Severe sheep injuries (*n* = 34), 17.4% of the most severe injuries (percent, 95% CI)	Recent horse injuries (*n* = 55), 23.4% of the most recent injuries (percent, 95% CI)	Severe horse injuries (*n* = 42), 21.5% of the most severe injuries (percent, 95% CI)
RIDDOR reportable	6.4% (1.3‒17.5)	9.8% (3.3‒21.4%)	6.5% (0.8‒21.4)	11.8% (3.3‒27.5)	9.1% (3.0‒20.0)	9.5% (2.7‒22.6)
Clinical status at time of injury						
Pre‐clinical	68.8% (53.8‒81.4)	70.6% (56.2‒82.5)	78.1% (60.0‒90.7)	88.2% (72.6‒96.7)	47.3% (33.7‒61.2)	61.9% (45.6‒76.4)
Clinical	31.3% (18.7‒46.3)	29.4% (17.5‒43.8)	21.9% (9.3‒40.0)	11.8% (3.3‒27.5)	52.7% (38.8‒66.4)	38.1% (23.6‒54.4)
Location of injury						
University premises	8.3% (2.3‒20.0)	5.9% (1.2‒16.2)	12.5% (3.5‒29.0)	8.8% (1.9‒23.7)	27.3% (16.1‒41.0)	16.7% (7.0‒31.4)
University farm	8.3% (2.3‒20.0)	3.9% (0.5‒13.5)	9.4% (2.0‒25.0)	5.9% (0.7‒19.7)	1.8% (0.0‒9.7)	
Farm animal practice		2.0% (0.0‒10.5)	3.1% (0.1‒16.2)	2.9% (0.1‒15.3)		
Equine hospital					18.2% (9.1‒30.9)	11.9% (4.0‒25.6)
Equine practice					5.5% (1.1‒15.1)	2.4% (0.1‒12.6)
Small animal hospital					1.8% (0.0‒9.7)	2.4% (0.1‒12.6)
External	4.2% (0.5‒14.3)	3.9% (0.5‒13.5)	3.1% (0.1‒16.2)	0	5.5% (1.1‒15.1)	2.4% (0.1‒12.6)
External university rotation					3.6% (0.4‒12.5)	
Visiting farm with university	4.2% (0.5‒14.3)	3.9% (0.5‒13.5)	3.1% (0.1‒16.2)		1.8% (0.0‒9.7)	2.4% (0.1‒12.6)
Visiting stable with university						
On extra‐mural studies	87.5% (74.8‒95.3)	90.2% (78.6‒96.7)	84.4% (67.2‒94.7)	91.2% (76.3‒98.1)	67.3% (53.3‒79.3)	81.0% (65.9‒91.4)
Farm	77.1% (62.7‒88.0)	74.5% (60.4‒85.7)	81.3% (63.6‒92.8)	88.2% (72.6‒96.7)	1.8% (0.0‒9.7)	
Farm animal practice	10.4% (3.5‒22.7)	15.7% (7.0‒28.6)			3.6% (0.4‒12.5)	2.4% (0.1‒12.6)
Equine yard or stables					47.3% (33.7‒61.2)	59.5% (43.3‒74.4)
Equine practice					14.5% (6.5‒26.7)	19.0% (8.6‒34.1)
Small animal practice			3.1% (0.1‒16.2)	2.9% (0.1‒15.3)		
Was any one present?	*n* = 44	*n* = 49	*n* = 29	*n* = 34	*n* = 53	*n* = 42
No‐one	22.7% (11.5‒37.8)	22.4% (11.8‒36.6)	20.7% (8.0‒39.7)	29.4% (15.1‒47.5)	24.5% (13.8‒38.3)	28.6% (15.7‒44.6)
Veterinarian	29.5% (16.8‒45.2)	26.5% (15.0‒41.1)	13.8% (3.9‒31.7)	5.9% (0.7‒19.7)	26.4% (15.3‒40.3)	23.8% (12.1‒39.5)
Owner of animal	22.7% (11.5‒37.8)	4.1% (0.5‒14.0)	17.2% (5.8‒35.8)	32.4% (17.4‒50.5)	1.9% (0.0‒10.1)	9.5% (2.7‒22.6)
Veterinary student	4.5% (0.6‒15.5)	24.5% (13.3‒38.9)	44.8% (26.5‒64.3)	17.6% (6.8‒34.5)	15.1% (6.7‒27.6)	4.8% (0.6‒16.2)
Other staff at the location	13.6% (5.2‒27.4)	12.2% (4.6‒14.8)	3.4% (0.1‒17.8)	2.9% (0.1‒15.3)	24.5% (13.8‒38.3)	33.3% (19.6‒49.6)
Farmer	6.8% (1.4‒18.7)	10.2% (3.4‒22.2)		11.8% (3.3‒27.5)		
Other university staff					3.8% (0.5‒13.0)	
What treatment was given?	*n* = 43	*n* = 49	*n* = 29	*n* = 34	*n* = 50	*n* = 42
None	18.6% (8.4‒33.4)	16.3% (7.3‒29.7)	24.3% (10.3‒43.5)	17.6% (6.8‒34.5)	18.0% (8.6‒31.4)	21.4% (10.3‒36.8)
Self‐administered first aid	60.4% (44.4‒75.0)	59.2% (44.2‒73.0)	69.0% (49.2‒84.7)	67.6% (49.5‒82.6)	64.0% (49.2‒77.1)	59.5% (43.3‒74.4)
Received first aid	4.7% (0.6‒15.8)	4.1% (0.5‒14.0)	6.9% (0.8‒22.8)	11.8% (3.3‒27.5)	2.0% (0.1‒10.7)	2.4% (0.1‒12.6)
Attendance at primary care practice	4.7% (0.6‒15.8)	6.1% (1.3‒16.9)		2.9% (0.1‒15.3)	4.0% (0.5‒13.7)	2.4% (0.1‒12.6)
Attendance at minor injury unit	7.0% (1.5‒19.1)	8.2% (2.3‒19.6)			6.0% (1.3‒16.6)	7.1% (1.5‒19.5)
Attendance at emergency department	4.7% (0.6‒15.8)	6.1% (1.3‒16.9)			4.0% (0.5‒13.7)	4.8% (0.6‒16.2)
Admission to hospital					2.0% (0.1‒10.7)	2.4% (0.1‒12.6)
Time to physically recover	*n* = 43	*n* = 49	*n* = 29	*n* = 34	*n* = 50	*n* = 42
<1 week	46.5% (31.2‒62.3)	34.7% (21.7‒49.6)	44.8% (26.5‒64.3)	32.4% (17.4‒50.5)	52.0% (37.4‒66.3)	42.9% (27.7‒59.0)
1‒2 weeks	27.9% (15.3‒43.7)	28.6% (16.6‒43.3)	34.5% (18.0‒54.3)	38.3% (22.2‒56.4)	28.0% (16.2‒42.5)	31.0% (17.6‒47.1)
2‒3 weeks	14.0% (5.3‒27.9)	14.3% (5.9‒27.2)	10.3% (2.2‒27.4)	11.8% (3.3‒27.5)		
3‒4 weeks	2.3% (0.1‒12.3)	6.1% (1.3‒16.9)	3.4% (0.1‒17.8)	2.9% (0.1‒15.3)	8.0% (2.2‒19.2)	14.3% (5.4‒28.5)
4+ weeks	9.3% (2.6‒22.1)	16.3% (7.3‒29.7)	6.9% (0.8‒22.8)	14.7% (5.0‒31.1)	12.0% (4.5‒24.3)	11.9% (4.0‒25.6)
Time off studies	*n* = 43	*n* = 49	*n* = 29	*n* = 34	*n* = 50	*n* = 42
None	100%	100%	100%	100%	96.0% (86.3‒99.5)	95.2% (83.8‒99.4)
<1 week					2.0% (0.1‒10.7)	2.4% (0.1‒12.6)
1‒2 weeks						
2‒3 weeks						
3‒4 weeks						
4+ weeks					2.0% (0.1‒10.7)	2.4% (0.1‒12.6)
Was the veterinary school informed of the injury?	*n* = 42	*n* = 49	*n* = 29	*n* = 34	*n* = 47	*n* = 42
Yes	9.5% (2.7‒22.6)	8.2% (2.3‒19.6)	13.8% (3.9‒31.7)	5.9% (0.7‒19.7)	21.3% (10.7‒35.7)	21.4% (10.3‒36.8)
Was any action taken	*n* = 39	*n* = 49	*n* = 29	*n* = 34	*n* = 47	*n* = 42
Yes	5.1% (0.6‒17.3)	4.1% (0.5‒14.0)			8.5% (2.4‒20.4)	9.5% (2.7‒22.6)
Don't know	7.7% (1.6‒20.9)	10.2% (3.4‒22.2)	10.3% (2.2‒27.4)	11.8% (3.3‒27.5)	6.4% (1.3‒17.5)	9.5% (2.7‒22.6)

Abbreviations: CI, confidence interval; RIDDOR, Reporting of Injuries, Diseases and Dangerous Occurrences Regulations.

Less than 20% of students received no medical treatment for their injuries, and more than 60% had first aid treatment. Almost 12% (11.7%) of recent and 14.3% of severe injuries required hospital treatment. Despite 25.6% of recent and 36.7% of severe injuries taking more than 2 weeks to physically recover, no students took time off their studies. Less than 10% of students informed their university about their injury. Only two actions subsequent of reporting were recorded, these were one farm and one university investigation.

Sheep‐related injuries predominately occurred while the students were involved in three key activities: restraint (55.2% of recent sheep injuries and 41.7% of most severe), lambing (13.8% and 16.7%) and drug administration (13.8% and 12.5%). More than one in five of these injuries occurred when the student was by themselves. Sheep‐related injuries were mainly the result of crushes (25.8% of recent and 23.5% of severe), headbutts (22.6% and 20.6%) and being knocked over (9.7% and 14.7%). Injuries were focused on hands (51.6% and 41.2%) and legs (19.4% and 17.6), but a concerning number of head injuries occurred (9.7% and 17.6%). The main injuries were bruising (51.6% and 44.1%) and lacerations (12.9% and 17.6%); however, there were concussions (3.2% and 5.9%), and 5.9% of most severe injuries were fractures. Seven percent (6.5%) of recent sheep‐related injuries and 11.8% of severe sheep‐related injuries were RIDDOR reportable injuries.

All sheep‐related head injuries were a result of being knocked over or head butted. These two quotes typify how these injuries were sustained:

I was trying to catch a ewe and when I finally caught hold of it, it pulled me head first into a steel girder

I was trying to catch a ewe for lambing and as I grabbed her she threw her head up and hit me in the face

Over 70% of students had first aid treatment, and no students attended a hospital or minor injury unit as a result of a sheep‐related injury. Despite 20.6% of recent and 29.4% of severe injuries taking more than 2 weeks to physically recover, no students took time off their studies. Less than 15% of students informed their university about their injury. No students reported that any action was taken by a university as a result of their sheep‐related injury.

### Horse‐related injuries

Around one‐quarter of the most recent injuries and over 20% of the most severe injuries were associated with horses. The most recent injuries were evenly split between clinical and pre‐clinical years, with the most common locations of injury being on an equine yard (47.3%), the university equine hospital (18.2%) and an EMS equine practice (14.5%) (Table 2). In contrast, the majority of the severe injuries occurred in pre‐clinical years, and primarily occurred on equine yards (59.5%) and EMS equine practice (19.0%). Injuries occurred while the students were involved in three key activities: restraining a horse (30.2% of recent injuries and 22.9% of severe injuries), during a clinical examination or procedure (17.0% and 20.0%) and while leading a horse (13.2% and 20.0%).

The majority of the restraining‐based injuries were for when a veterinarian was performing a procedure on the horse, for example:

I was holding a young horse with a headcollar and lead rope while a vet administered an intramuscular injection. The horse spooked at the injection and barged into me pressing me against the stable wall as it went through the stable door

All clinical examinations and procedure‐based injuries were when the student was examining, or holding the x‐ray plate by, a distal limb.

After showing where the horse where our hands were, I inserted the x‐ray plate medial to the stifle and was kicked on the leg

Around one‐quarter of all equine‐related injuries occurred when the student was by themselves. Injuries were mainly the result of kicks (32.7% of recent and 40.5% of severe), bites (25.5% and 11.9%) and crushes (23.6% and 23.8%). The distribution of body parts injured was dispersed: leg (36.4% and 38.1%), arm (16.4% and 9.5%), foot (14.5% and 11.9%) and hand (10.9% and 16.7%). Heads were involved in 3.6% and 7.1% of injuries, most of these were the result of kicks. Injuries were overwhelming bruises (74.5% and 69.0%), but there were fractures (10.9% and 11.9%) and dislocations (5.6% and 2.4%). Nine percent (9.1%) of recent injuries and 9.5% of severe injuries were RIDDOR reportable injuries.

Around one in five students received no treatment with the majority self‐administering first aid. Twelve percent of recent and 14.3% of severe injuries required hospital treatment. Despite 20% of recent and 26.2% of severe injuries taking more than 2 weeks to physically recover, only a very small percentage of students took time off their studies. Around one in five students informed their university about their injury. Only four actions or behaviour changes subsequent to reporting an incident were recorded. These included warning signs provided for specific horses, new pathways developed from yards to paddocks, and a referral to disability services.

### Companion animal

Over one‐third of the most recent injuries and one‐quarter of the most severe injuries were associated with cats and dogs. These overwhelmingly occurred in clinical years, except for severe injuries caused by dogs where there was an even split between pre‐clinical and clinical years (Table 3). The majority of injuries occurred on EMS placements, in particular in small animal practices. Cat‐related injuries predominately occurred while students were involved in four key activities: restraint (51.3% of recent injuries and 50.0% of severe injuries), clinical examinations (10.3% and 8.3%), taking a cat in or out of a kennel (10.3% of recent injuries) and administering oral medication (7.7% and 12.5%). The majority of the time, a veterinarian or veterinary nurse was present when the student was injured. Cat‐related injuries were mainly the result of scratches (55.3% and 62.1%) and bites (44.7% and 37.9%). Three key body parts were injured in cat‐related incidents: hands (46.8% and 51.7%), arms (44.7% and 37.9%) and heads (6.4% and 10.3%). The injuries were lacerations (53.2% and 51.7%) and puncture wounds (38.3% and 37.9%). There were no fractures or concussions. No cat‐related injuries were RIDDOR reportable injuries.

**TABLE 3:** Context of injuries to veterinary students caused by companion animals.

	Recent cat injuries (*n* = 47), 20.0% of the most recent injuries (percent, 95% CI)	Severe cat injuries (*n* = 29), 14.9% of the most severe injuries (percent, 95% CI)	Recent dog injuries (*n* = 33), 14.0% of the most recent injuries (percent, 95% CI)	Severe dog injuries (*n* = 21), 10.8% of the most recent injuries (percent, 95% CI)
RIDDOR	0	0	6.1% (0.7‒20.2)	4.8% (0.1‒23.8)
Clinical status at time of injury				
Pre‐clinical	19.1% (9.1‒33.3)	13.8% (3.9‒31.7)	30.3% (15.6‒48.7)	47.6% (25.7‒70.2)
Clinical	80.9% (66.7‒90.9)	86.2% (68.3‒96.1)	69.7% (51.3‒84.4)	52.4% (29.8‒74.3)
Location of injury				
University premises	4.3% (0.5‒14.5)	3.4% (0.1‒17.8)	15.2% (5.1‒31.9)	4.8% (0.1‒23.8)
Small animal hospital	4.3% (0.5‒14.5)	3.4% (0.1‒17.8)	9.1% (1.9‒24.3)	0
Small animal practice	0	0	6.1% (0.7‒20.2)	0
Equine practise	0	0	0	4.8% (0.1‒23.8)
External	0	3.4% (0.1‒17.8)	0	0
External university rotation	0	3.4% (0.1‒17.8)	0	0
On extra‐mural studies	95.7% (85.5‒99.5)	93.1% (77.2‒99.2)	84.8% (68.1‒94.9)	95.2% (76.2‒99.9)
Small animal practice	87.2% (74.3‒95.2)	86.2% (68.3‒96.1)	66.7% (48.2‒82.0)	57.1% (34.0‒78.2)
Kennel or cattery	8.5% (2.4‒20.4)	0	15.2% (5.1‒31.9)	33.3% (14.6‒57.0)
Farm	0	0	3.0% (0.1‒15.8)	4.8% (0.1‒23.8)
Equine yard or stable	0	6.9% (0.8‒22.8)	0	0
Was any one present?	*n* = 47	*n* = 29	*n* = 28	*n* = 21
No‐one	31.9% (19.1‒47.1)	20.7% (8.0‒39.7)	21.4% (8.3‒41.0)	19.0% (5.4‒41.9)
Veterinarian	57.4% (42.2‒71.7)	65.5% (45.7‒82.1)	53.6% (33.9‒72.5)	38.1% (18.1‒61.6)
Veterinary nurse	8.5% (2.4‒20.4)	10.3% (2.2‒27.4)	10.7% (2.3‒28.2)	9.5% (1.2‒30.4)
Other staff at the location	2.1% (0.1‒11.3)	0	3.6% (0.1‒18.4)	14.3% (3.0‒36.3)
Owner of animal	0	3.4% (0.1‒17.8)	7.1% (0.9‒23.5)	4.8% (0.1‒23.8)
Veterinary student	0	0	0	9.5% (1.2‒30.4)
Farmer	0	0	3.6% (0.1‒18.4)	4.8% (0.1‒23.8)
What treatment was given?	*n* = 38	*n* = 29	*n* = 28	*n* = 21
None	2.6% (0.1‒13.8)	3.4% (0.1‒17.8)	3.6% (0.1‒18.4)	0
Self‐administered first aid	63.1% (46.0‒78.2)	58.6% (38.9‒76.5)	64.3% (44.1‒81.4)	52.4% (29.8‒74.3)
Received first aid	7.9% (1.7‒21.4)	13.8% (3.9‒31.7)	14.3% (4.0‒32.7)	14.3% (3.0‒36.3)
Attendance at primary care practice	13.2% (4.4‒28.1)	13.8% (3.9‒31.7)	3.6% (0.1‒18.4)	4.8% (0.1‒23.8)
Attendance at minor injury unit	10.5% (2.9‒24.8)	6.9% (0.8‒22.8)	10.7% (2.3‒28.2)	14.3% (3.0‒36.3)
Attendance at an emergency department	2.6% (0.1‒13.8)	3.4% (0.1‒17.8)	3.6% (0.1‒18.4)	14.3% (3.0‒36.3)
Admission to hospital	0	0	0	0
Time to physically recover	*n* = 38	*n* = 29	*n* = 28	*n* = 21
<1 week	68.4% (51.4‒82.5)	62.1% (42.3‒79.3)	71.4% (51.3‒86.8)	42.9% (21.8‒66.0)
1‒2 weeks	18.4% (7.7‒34.3)	31.0% (15.3‒50.8)	14.3% (4.0‒32.7)	28.6% (11.3‒52.2)
2‒3 weeks	10.5% (2.9‒24.8)	3.4% (0.1‒17.8)	7.1% (0.9‒23.5)	9.5% (1.2‒30.4)
3‒4 weeks	2.6% (0.1‒13.8)	3.4% (0.1‒17.8)	7.1% (0.9‒23.5)	9.5% (1.2‒30.4)
4+ weeks	0	0	0	9.5% (1.2‒30.4)
Time off studies	*n* = 38	*n* = 29	*n* = 28	*n* = 21
None	100%	100%	100%	100%
Was the veterinary school informed of the injury?	*n* = 37	*n* = 29	*n* = 28	*n* = 21
Yes	8.1% (1.7‒21.9)	3.4% (0.1‒17.8)	28.6% (13.2‒48.7)	23.8% (8.2‒47.2)
Was any action taken	*n* = 37	*n* = 29	*n* = 28	*n* = 21
Yes	2.7% (0.1‒14.2)	3.4% (0.1‒17.8)	0	0
Don't know	21.6% (9.8‒38.2)	17.2% (5.8‒35.8)	25.0% (10.7‒44.9)	23.8% (8.2‒47.2)

Abbreviations: CI, confidence interval; RIDDOR, Reporting of Injuries, Diseases and Dangerous Occurrences Regulations.

The majority of students received medical treatment for their cat‐related injuries, with more than 60% receiving first aid treatment. Thirteen percent (13.1%) of recent and 10.3% of severe injuries required hospital treatment. Despite 13.2% of recent and 6.8% of severe cat‐related injuries taking more than 2 weeks to physically recover, no students took time off their studies. Fewer than 10% of students reported cat‐related injuries to their university, compared with about one‐quarter for dog‐related injuries.

Students were injured by dogs during many diverse activities; however, 41.3% of recent injuries and 43.8% of severe injuries occurred when restraining a dog. The majority of the time, a veterinarian or veterinary nurse was present when the student was injured. Dog‐related injuries were mainly the result of bites (54.5% and 71.4%), scratches (27.3% and 9.5%) and head butts (9.5% of severe injuries). Three key body parts were injured: hands (57.6% and 66.7%), arms (27.3% and 19.0%) and legs (9.1% and 9.5%). Dog‐related injuries were lacerations (27.3% and 28.6%), puncture wounds (30.3% and 28.6%) and bruising (21.2% and 23.8%). There were three fractures and no concussions. Six percent (6.1%) of recent dog‐related injuries and 4.8% of severe injuries were RIDDOR reportable injuries.

Less than 4% of students received no medical treatment for their dog‐related injuries, and more than half had first aid treatment. Fourteen percent (14.3%) of recent and 28.3% of severe injuries required hospital treatment. Despite 14.2% of recent and 28.5% of severe injuries taking more than 2 weeks to physically recover, no students took time off their studies. Around one‐quarter of students informed their university about their dog‐related injury. Students recalled that no actions were taken subsequent of reporting.

### Needlestick injuries

Almost 5% of recent injuries (4.7%, 95% CI 2.4‒8.3) and 3.6% (95% CI 1.5‒7.3) of their most severe injuries were needlestick injuries. The main mechanisms involved were restraining the animal for a blood sample or intravenous catheter placement (27.8%), intra‐muscular injection (22.2%), recapping a needle (16.7%) and having a needle in the pocket (11.1%). In only one instance was the drug in the syringe mentioned, namely, pentobarbital.

### Qualitative content analysis

The majority of students did not experience emotional or mental effects resultant of their injuries (Table 4). When effects were mentioned the most prevalent themes were an increased anxiety or fear around a specific species and being more cautious. For example:

**TABLE 4:** Content analysis of behaviours reported by veterinary students subsequent to their most recent, and their most severe, animal‐related injuries.

Themes	Cattle		Sheep		Horse		Cat		Dog	
	Recent	Severe	Recent	Severe	Recent	Severe	Recent	Severe	Recent	Severe
Did you experience any emotional or mental effects resultant of the injury?	*N* = 43	*N* = 49	*N* = 29	*N* = 34	*N* = 50	*N* = 42	*N* = 38	*N* = 29	*N* = 28	*N* = 21
None	62.8%	63.3%	55.2%	73.5%	62.0%	59.5%	78.9%	82.3%	78.6%	71.4%
Increased anxiety or fear	20.9%	20.4%	6.9%	5.9%	18.0%	19.0%	7.9%	6.9%	14.3%	28.6%
More cautious around the animal	2.3%	6.1%	13.8%	2.9%	12.0%	14.3%	7.9%	6.9%	3.6%	0
Won't work with that species again	7.0%	4.1%	0	2.9%	2.0%	2.4%	2.6%	0	0	0
Confidence knocked	4.7%	4.1%	0	0	2.0%	4.8%	0	0	3.6%	4.8%
Embarrassment	2.3%	2.0%	6.9%	2.9%	4.0%	2.4%	2.6%	3.4%	0	0
‘It made me stronger’	4.7%	2.0%	0	5.9%	2.0%	2.4%	0	0	0	0
Why did you not take time off from your studies?	*N* = 42	*N* = 49	*N* = 29	*N* = 34	*N* = 44	*N* = 40	*N* = 37	*N* = 28	*N* = 28	*N* = 21
A minor injury	61.9%	57.1%	75.9%	76.5%	59.1%	52.5%	78.4%	82.1%	75.0%	61.2%
Felt no need as during their EMS period	11.9%	16.3%	13.8%	8.8%	13.6%	17.5%	10.8%	3.6%	17.9%	33.3%
Tight timetabling	14.3%	20.4%	3.4%	11.8%	18.2%	17.5%	2.7%	0	0	0
Could still function, just!	11.9%	8.5%	3.4%	0	11.4%	15.0%	5.4%	10.7%	3.6%	4.8%
Didn't want to get in trouble with university or EMS provider	7.1%	4.1%	6.9%	2.9%	15.9%	15.0%	0	3.6%	0	0
‘Just get on with it!’	7.1%	4.1%	0	2.9%	4.5%	10.0%	2.7%	3.6%	3.6%	0
Why did you choose not to report the incident?	*N* = 36	*N* = 42	*N* = 25	*N* = 32	*N* = 38	*N* = 40	*N* = 24	*N* = 19	*N* = 19	*N* = 21
Only minor/wasn't serious enough	61.1%	47.6%	68.0%	59.4%	65.8%	52.5%	79.2%	78.9%	68.4%	33.3%
Unaware needed reporting	13.9%	23.8%	16.0%	25.0%	15.8%	17.5%	20.8%	21.1%	36.8%	28.6%
Didn't want to make a fuss	13.9%	19.0%	0	0	15.8%	17.5%	8.3%	15.8%	10.5%	9.5%
Too much effort (what's the benefit?)	16.7%	26.2%	8.0%	12.5%	10.5%	15.0%	4.2%	0	5.3%	4.8%
Felt silly	8.3%	7.1%	12.0%	6.3%	5.3%	15.0%	8.3%	10.5%	0	4.8%
It's just one of those things/part of the course	2.8%	4.8%	4.0%	12.5%	5.3%	10.0%	4.2%	5.3%	5.3%	4.8%
Have you changed your behaviour around the animal species involved in the injury?	*N* = 43	*N* = 39	*N* = 43	*N* = 34	*N* = 50	*N* = 42	*N* = 38	*N* = 29	*N* = 28	*N* = 21
No	32.6%	41.0%	37.2%	55.9%	44.0%	47.6%	44.7%	37.9%	39.3%	28.6%
Caution/less confident	34.9%	41.0%	4.7%	8.8%	16.0%	11.9%	7.9%	10.3%	21.4%	33.3%
Won't do procedure/avoid animal	4.7%	5.1%	0	2.9%	8.0%	7.1%	5.3%	6.9%	7.1%	4.8%
Do procedure differently	4.7%	20.5%	9.3%	11.8%	8.0%	7.1%	7.9%	6.9%	0	4.8%
Improve handling/restraint	11.6%	10.3%	4.7%	2.9%	0	4.8%	26.3%	20.7%	28.6%	28.6%
Use personal protective equipment	0	2.7%	0	0	16.0%	16.7%	0	0	0	0
Denial that did anything wrong	9.3%	5.1%	7.0%	2.9%	2.0%	0	5.3%	6.9%	7.1%	9.5%
Improved awareness of surroundings	9.3%	17.9%	4.7%	14.7%	8.0%	9.5%	0	0	0	0

Abbreviation: EMS, extra‐mural studies.

Fear of getting kicked by cows and anxiety around cows in general—fractured hand, cow kick

Fear of being bitten again—hand laceration, dog bite

Some livestock‐ and horse‐related injuries led students to report wanting to avoid these species professionally:

I only know I won't be working with cows, thank you very much—bruised hand, cow crush

Less confidence with horses. I already wasn't equine inclined but I am experienced with handling and riding horses. However, this experience made me realise how dangerous horses can be especially in a veterinary context and it has made me more nervous to give horses injections. It has definitely confirmed I don't want to work in equine practice—bruised arm, horse crush

Others stated that their confidence was knocked, and that becoming injured could be a source of embarrassment:

Embarrassment for getting upset in front of the vet (got teary due to the shock of the situation at the time not due to pain)—face laceration, cat scratch

I got very bad feedback from the EMS provider, I believe they were probably scared of the fact I was hurt so wanted to make it seem as though I was incompetent. This affected my confidence for at least a year—hand laceration, dog bite

Some students, although only those injured by livestock or horses, stated that they felt the injuries made them stronger and were beneficial to them:

It made me stronger. Bumps and bruises are part of the working farm environment, even if you get bowled over once in a while—bruised leg, cow trampling

The main reason that the majority of students gave for not taking time off their studies was that they had only received a minor injury. Based on the injury description this was accurate for most cases. A proportion did not view EMS as part of their studies, and so time off from EMS was not viewed as time off their studies to recover.

I attended placement in a holiday and so I was fully healed by the time I started back at uni—laceration of arms and head, pig goring

Many, especially those injured by cattle and horses, felt they could not take time off as there was no resource allocation to reschedule their placements:

I knew that if I took time off of the EMS placement I'd have to resist it and I didn't have the time or money as it cost me £40 a day to travel there—bruised arm, cow kick

I felt I had to sort of grin and bear it because I worry that you can't really take time off veterinary. We have so much EMS to fit in and attendance of our lectures/practicals is very closely monitored—abdominal bruising, multiple horse kicks

Some students, despite having relatively serious injuries, felt that they could still function enough to work and so did not take time off:

Didn't inhibit me too much and I've broken fingers before so didn't bother me too much—fractured hand and fingers, cattle crush

Some, especially those injured by horses and livestock, felt that the injury could get them in trouble with either their university or EMS provider:

Was told by host practice if I left the placement to recover I would not be welcome to return—fractured skull, horse kick

I needed to complete the EMS placement. I was worried my supervisor wouldn't have signed my placement if I took a time off—bruised abdomen, trampled by horse

Many students described feeling that they needed to be tough and ‘get on with it’;

You just have to get on with it. A bruise or a cut shouldn't stop you from being able to do what you have to—bruised head, sheep head butt

I was determined to push through and just deal with it, and I did—friction burn of hand, escaped horse

Felt like I had to show I was tough and walk it off—sprained leg, knocked over by horse

Most students who did not report injuries felt they were not serious enough, although perceptions of ‘severity’ were influenced by reporting concerns and an expectation of injury:

It was not serious, I would not want a report to impact my studies or the practice I was at just because a cat had good aim. I recognise that as an everyday hazard of the job—bruised hand, cat scratch

Many described being unaware that reporting was needed. Some did not want to ‘make a fuss’, while others felt it was too onerous and could not see any benefit to reporting, compared with the perceived negative impact of raising concerns:

Can't be arsed with paperwork, seems a bit dramatic, I didn't lose a finger. I'd probably be laughed at and they wouldn't know how to report. The outcome wouldn't benefit me so why would I?—puncture wound to hand, fox bite

Not necessary—protocol is idiotic—arm pain, dog bite

What difference would it make other than making me fill out paperwork—jaw fracture, horse kick

Some felt there could be personal emotional repercussions of being made to feel stupid, embarrassed or not taken seriously:

I didn't want them to force me to take time off—foot fracture, cow crush

It was a one‐vet, one‐nurse practice. This vet is very unprofessional and said to ‘grow up, it happens’ and barely gave me the time to remove the nail (which was hanging off) and wash and bandage it before asking for help with the next animal. The nurse was too afraid to speak up in front of the vet—fingernail avulsion, dog headbutt

Many felt that injuries were just part and parcel of being a veterinarian and so they did not need reporting:

It was only a bruise and a bit of an occupational hazard!—leg bruise, sheep kick

Risks of doing TPR, everyone gets kicked at some point—leg bruise, horse kick

More than one‐third of students stated that they would not change their behaviour around the species involved in their injury, and others stated that the injury was inevitable and could not be avoided:

No, I was just unlucky—leg bruise, cow kick

Of those that did say they would change their behaviour, many said that they would exercise caution or be less confident around those animals, while other would use greater restraint, be more aware of their surroundings, do the procedure differently, and wear personal protective equipment.

More cautious around handling dogs, will muzzle dogs more often—leg laceration, dog bite

Would perhaps be more likely to wear a helmet around the yard as well as riding. It could easily have been my head trampled instead of my legs—sprained leg, knocked over by horse

Will refuse to work with cows if there is no proper restraint. From now on, crush or nothing—bruised hand, cow crush

Some individuals stated that they would just avoid that species in their future careers:

There was nothing I could have done to avoid the injury so there is nothing I can change other than just not working with them—bruised arm, cow kick

## DISCUSSION

This study explored veterinary student injuries and resultant behaviours during formal training, an area that has received little prior attention. We found that levels of injury are higher than previous audits of veterinary school injury records,^5^ as students reported being unwilling to use formal reporting systems. We report a concerningly high level of injury, with almost half of students being injured by an animal during their degree, and an annual prevalence of around 35%. Many students experienced multiple injuries throughout their studies.

This study found that the risk of injury is highest on EMS placements. This is likely to have occurred because students are in diverse placement types and locations, with variable safety standards and supervision. Extra‐mural studies placements provide a challenge to the veterinary schools as students are not under their direct supervision at this point,^24^, ^25^ and safety practices may inevitably vary from practice to practice. A consultation process that explored provider and student concerns over EMS placements in 2010 did not find that safety was a concern for either group, which could suggest that health and safety management is not overtly discussed.^24^ Nevertheless, these placements are an integral part of the veterinary curriculum and highly beneficial for student learning^26^; as such, it is vital that students are given additional support during them in order to recognise and avoid risk and to empower students to report placements where health and safety standards are low. Furthermore, future research should focus on exploring handling techniques that simultaneously reduce human injury and uphold stringent animal welfare standards.

We found that accident reporting is low overall, with few students reporting their injuries. If one were to extrapolate out our findings to the whole student population, at least 2554 injuries would occur annually. An audit of five UK veterinary schools found on average 58 injuries reported annually.^5^ This highlights that currently reporting systems do not capture the full extent of the injuries that students are experiencing, particularly on EMS placements. This underestimation could inhibit universities’ abilities to make meaningful changes to student safety and thus warrants attention. Students in the UK are not legally required to report every injury to their university, but institutions typically require reporting for incidents that occur on campus or during university‐organised activities so they can meet their own health and safety legal obligations.^20^ Injuries that happen outside university activities (such as EMS) generally carry no obligation for students to report them. However, many veterinary schools see that students are under their care and have a moral obligation to their safety when on placements and so encourage students to report accidents, incidents or near misses. Reasons that students gave for not reporting reflect those frequently found in the veterinary profession; lack of knowledge of reporting structures; lack of confidence in meaningful change as a result of reporting; and disinclination to draw attention to the event, sometimes for fear of being blamed.^3^, ^4^ It is unclear whether the students are mirroring or embracing the culture of the veterinary profession or whether these behaviours are established before attending university. This warrants further exploration.

This culture of under‐reporting could lead to long‐term habits that persist into professional practice, reducing the visibility of workplace risks and limiting prevention opportunities.^27^, ^28^ In other industries, effective interventions that encourage reporting have focused on improved psychological safety, dialogue and feedback between accident reporters and management, leading to visible improvements to systems on the basis of reports.^29^, ^30^, ^31^ Importantly, a meta‐analysis of behavioural interventions used to improve safety across industries found strong evidence that initiatives that were aimed at the industry, culture or group level were more successful than those aimed at individuals.^32^ Applying a similar approach at veterinary schools could foster a culture that encourages transparent reporting while maintaining anonymity and emphasises the role that reporting has in improving student safety and animal handling practices.

Few students sought medical treatment beyond self‐administered first aid or took time off their studies to recover; even when the injury was severe enough to warrant these behaviours. Students often attributed this to beliefs that injury was an expected part of veterinary training, that ‘toughness’ was valued, and to limited resources (i.e., time or funding) to pause studies or repeat placements. This appears particularly relevant for younger individuals, who may feel their input has limited influence on safety improvements, which is especially common among young women in other workplaces.^33^ Females make up the majority of veterinary school students and it has been previously reported that gender stereotypes exist in the veterinary industry around gendered work; for example, that females will be better suited to small animal settings and that farm work is ‘masculine’.^34^ Exploring the impacts of gender on injury prevalence and perception and the need to display ‘toughness’ in future studies could yield valuable insight. The culture of limited injury care and limited time off work reflects very closely that of the veterinary profession.^3^, ^4^ This is shaped by heavy workloads and academic demands, which could discourage rest or recovery, as well as by a culture of stoicism and presenteeism that normalises working through pain or injury. Over time, this normalisation of risk could reinforce under‐reporting and under‐appreciating the seriousness of injuries, embedding habits that students are likely to carry into their professional careers.

The prevalent events preceding cattle‐related injuries (drug administration, herding, and milking) were primarily reflective of the students’ activities during pre‐clinical EMS. From the responses it is unclear why injuries during these events were more prevalent and further exploration is needed. The injuries received were relatively less traumatic than what is reported in the literature, which predominately focuses on individuals attending emergency departments or admissions to hospital for cattle‐related injuries (less than 10% of students within our data).^35^, ^36^, ^37^, ^38^, ^39^ This is likely due to the lack of studies describing cattle‐related injuries not requiring hospital treatment. Sheep‐related injuries were reflective of common activities performed during EMS placements (i.e., restraint, lambing and drug administration). For both cattle‐ and sheep‐related injuries, over 20% of students were injured while alone, which highlights concerns around supervision during EMS placements. This underscores the need to consider how best to safeguard students on farms.

Horse‐related injuries occurred while the students were restraining or leading a horse, or when examining a distal limb. Students received more bites than anticipated, and fewer head injuries.^2^, ^4^ The bites are likely reflective of the frequency of close‐hand tasks, such as restraint and leading horses, which compared to students, veterinarians are less likely to perform. Conversely, veterinarians are more likely to perform clinical tasks that increase the risk of a head injury, such as distal limb examinations and procedures,^2^ with students often positioned as handlers. When head injuries did occur in this survey, they were often during a distal limb examination. There were a number of fractures and dislocations, highlighting that equine EMS placements still pose a substantial risk of severe injury. Some students described subsequent efforts to better understand horse behaviour, recognising that improved awareness of equine body language may help anticipate and prevent risky situations.^40^ While reflection is valuable for personal learning, reliance on individual adaptation alone risks placing responsibility on students rather than addressing systemic safety issues, which is one of the least successful approaches to behaviour change.^32^

Cat‐related injuries were most commonly sustained during restraint, clinical examination, taking a cat in/out of a kennel and administration of oral medications. These are all routine tasks that a student would undertake at a university or on an EMS placement. While injuries were generally less severe than those associated with large animals, cat bites frequently resulted in puncture wounds to the hands and wrists. Such injuries are clinically significant, as cat bites can rapidly lead to serious infections, and current recommendations state that all cat bites should be assessed at a hospital and treated with antibiotics.^41^ However, only around one in 10 students sought hospital care following a bite. Students’ reluctance to seek care may reflect a professional culture of minimising so‐called minor injuries, norms that are systemic within the veterinary profession.^3^, ^14^ This underscores the need to address cultural factors within veterinary training to foster a safety‐oriented mindset from the outset of professional practice.

Injuries caused by dogs showed considerable contextual variation, with no clear trends across activities. The majority of incidents involved bites, reflecting the well‐documented risk dogs pose in veterinary practice.^3^, ^6^, ^42^ However, unlike previous research that has highlighted a notable proportion of dog bites to heads,^3^, ^5^ this study found relatively few such cases. This discrepancy may be explained by the supervised nature of student involvement, where students are often tasked with handling or restraint rather than performing procedures that place the head and upper body near the animal. Mirroring our findings, other research exploring occupational risks of dog bites across different workplaces, including veterinarians and postal workers, found that participants altered their own individual behaviour to avoid risk and experienced fear after being bitten, but did not trust that reporting incidents would lead to change.^43^ This study suggests that victim blaming is common in relation to dog bites, but unhelpful because it reduces the likelihood of cultural or organisational change, which could have far‐reaching impacts.

The qualitative content analysis revealed the extent to which culture plays a role in accident experience, behaviour and reporting: it was clear that students expected injuries to occur as part of their training, including severe injuries. As such, they described feeling a need to limit reporting and avoid being seen to ‘make a fuss’ in the face of placement providers and other veterinarians. This may be at least partly caused by the tendency towards blaming the injured party,^32^, ^42^ as well as the fear of having marks downgraded. The resulting culture of bravado around injury has the potential to increase levels of harm, for example by students feeling that they are implicitly expected to put themselves in risky situations, and by reducing help‐seeking behaviour after incidents occur. Research examining injury risk factors and strategies for improving safety emphasises the need for cultural change, including shifts in managerial practices and organisational norms.^27^, ^28^, ^32^ Given that graduate veterinary surgeons have been shown to accept injury as an inherent part of their work and that the likelihood of injury is influenced by local workplace culture,^3^, ^4^ it is important to examine how culture operates across the profession.

The evidence presented here identifies a poor safety culture in the veterinary student population, which should be of serious concern for the profession. We do not know where this culture begins; whether before or during veterinary education, on placement or at university sites. Some students may already carry attitudes shaped by prior experiences with animals, such as work‐experience, where risk‐taking or ‘getting the job done’ was rewarded. Once at veterinary school, these attitudes could be reinforced if safety protocols are inconsistently enforced or if students witness peers or instructors normalising minor injuries or risky practices. Extra‐mural studies placements may further expose students to high‐pressure and fast‐paced environments, where time constraints, hierarchical dynamics, and the prioritisation of patient care over personal safety can encourage shortcuts or silence around reporting injuries. Collectively, these experiences may influence how students come to understand which behaviours are tolerated, which risks seem acceptable, and whose voices are heard in discussions about safety. Further research is needed to understand whether and how such early experiences might shape a culture in which safety is undervalued, and preventable harm becomes routine.

### Limitations

This cross‐sectional study is limited by its inability to establish causality and the potential for self‐selection bias inherent in non‐probability sampling. The self‐reported data may be affected by recall bias^44^; for example, students may have felt more compelled to respond to the survey if they had experienced an injury or accident during their studies, even though steps were taken to mitigate this risk (the advertisement was designed to encourage participation for those who both had and had not experienced injury or illness). Reassuringly, the demographics of participants reflected that of veterinary students in the UK, suggesting a broadly representative sample. Future research should utilise longitudinal designs and probability sampling to improve generalisability or use interviews or focus groups to gain deeper qualitative insight.

## CONCLUSIONS

This survey provides valuable insights for the veterinary profession into the realities students face around injury and injury‐related behaviours during their training. We identify concerningly high levels of injury and severe injury, much of which is under‐reported, alongside a culture of injury acceptance and expectation. The activity‐specific risks highlighted in our data should assist universities and placement providers in strengthening, training, supporting and reporting structures. However, the expectation of injury and the tendency to continue working while injured remain particular concerns, and organisations may benefit from drawing on lessons from other sectors that have successfully shifted cultures around risk management and reporting.

## AUTHOR CONTRIBUTIONS

*Conceptualisation, methodology, analysis and writing—review and editing*: John S.P. Tulloch and Tamzin Furtado. *Methodology, analysis and writing—original draft*: Lois Kennedy. *Conceptualisation and writing—review and editing*: Gina Pinchbeck.

## CONFLICTS OF INTEREST

The authors declare they have no conflicts of interest.

## ETHICS STATEMENT

The study received ethical approval from the University of Liverpool Veterinary Research Ethics Committee (VREC1103).

## Data Availability

The raw data for this study are unlimited free‐text responses to survey questions, which contain potentially identifying and sensitive patient information. We are unable to share these data publicly because of restrictions by the University of Liverpool Veterinary Research Ethics Committee as participants did not consent to sharing their data outside of the study team. Relevant, de‐identified excerpts of the transcripts are included in the paper. Requests for additional information can be sent to the University of Liverpool Veterinary Research Ethics Committee at ivesethics@liverpool.ac.uk.
